# Efficacy of behavioural interventions for transport behaviour change: systematic review, meta-analysis and intervention coding

**DOI:** 10.1186/s12966-014-0133-9

**Published:** 2014-11-28

**Authors:** Bronia Arnott, Lucia Rehackova, Linda Errington, Falko F Sniehotta, Jennifer Roberts, Vera Araujo-Soares

**Affiliations:** Institute of Health and Society, Newcastle University, Newcastle-Upon-Tyne, UK; Department of Economics, University of Sheffield, Sheffield, UK

## Abstract

**Background:**

Reducing reliance on motorised transport and increasing use of more physically active modes of travel may offer an opportunity to address physical inactivity. This review evaluates the evidence for the effects of behavioural interventions to reduce car use for journeys made by adults and codes intervention development and content.

**Methods:**

The review follows the procedure stated in the registration protocol published in the PROSPERO database (registration number CRD42011001797). Controlled studies evaluating behavioural interventions to reduce car use compared with no interventions or alternative interventions on outcome measures of transport behaviours taken in adult participants are included in this review. Searches were conducted on all records in Applied Social Sciences Index and Abstracts (ASSIA), Ovid Embase, Ovid Medline, Ovid PsycInfo, Scopus, Sociological Abstracts, Transportation Research Information Service (TRIS), Transportation Research International Documentation (TRID), and Web of Science databases. Peer reviewed publications in English language meeting the inclusion criteria are eligible. Methodological quality is assessed using the Cochrane Risk of Bias Tool. Interventions are categorised in terms of behavioural frameworks, theories and techniques.

**Results:**

15 full text articles are included, representing 13 unique studies, with 4895 participants and 27 intervention arms. Risk of bias across the review is appraised as considerable due to the unclear methodological quality of individual studies. Heterogeneity of included studies is considerable. Meta-analyses reveal no significant effect on reduction of frequency of car use or on increasing the proportion of journeys by alternative, more active modes of transport. There is insufficient data relating to alternative outcomes such as distance and duration which may have important health implications. Interventions were top-down but could not be described as theory-based. Intervention efficacy was associated with the use of a combination of information provision and behavioural regulation techniques. There was a lack of consideration of opportunity for change and behaviour in context.

**Conclusions:**

There is no evidence for the efficacy of existing behavioural interventions to reduce car trips included in this review. The evidence for efficacy of behavioural interventions to decrease distance and duration of car journeys is limited and inconclusive. Overall the evidence is highly heterogeneous and is at considerable risk of bias. Future research should investigate alternative behavioural interventions in high quality, controlled studies informed by existing evidence, theory, and viewers of potential users. Future intervention studies should increase scientific rigour, include objective outcome measures, and incorporate thorough evaluations as standard.

**Electronic supplementary material:**

The online version of this article (doi:10.1186/s12966-014-0133-9) contains supplementary material, which is available to authorized users.

## Background

Increasing active travel and reducing the reliance on motorised transport are increasingly high on the health agenda [1,2]. Driving a car is defined as a sedentary behaviour; a distinct class of behaviours characterised by limited physical movements and low energy expenditure [3]. Increased levels of sedentary behaviour are associated with a range of health risks including Type 2 Diabetes [4]. There is evidence of a distinct, dose response relationship between sedentary lifestyles and premature mortality [5].

Engagement in alternative, more active travel behaviours (walking or cycling) for all or parts of regular journeys could assist individuals in achieving current national [6] and international [7] activity level guidelines. In 2008 around 39% of men and 29% of women in England reported meeting the minimum physical activity recommendations of the Chief Medical Officer [8]. However, objective measures of activity, such as accelerometers, suggest much lower adherence to guidelines with only 6% of males and 4% of females achieving minimum recommendations [8].

In 2011 in England and Wales, 64% of trips were made by private motorised vehicles, 23% by walking and 2% by cycling [9]. Interventions to decrease car journeys and increase active travel behaviours could help to reduce time spent being sedentary and increase levels of physical activity.

Interventions to promote transport behaviour change can be divided into those with structural or behavioural components, or those with a combination of both. Structural interventions involve modification of the physical environment and the physical choice architecture. These may include road/fuel pricing, planning bus and cycle lanes, and pedestrianisation of city centres. Behavioural interventions target communities and individuals with methods directed at changing affects, beliefs and attitudes about behavioural options or supporting self-regulation [10]. They can include providing feedback, facilitating social comparison, and providing economic incentives.

At present there is limited evidence on how to promote transport behaviour change. A review by Ogilvie and colleagues [11] examined which interventions are effective in promoting a population shift from using cars towards walking and cycling in 2004. This review concluded that interventions could see a 5% shift in population level transport behaviours in motivated sub-groups of individuals. However, this review did not consider uptake of other alternatives to car driving, such as public transport use, which may also decrease physical inactivity. A recent review of the car reduction evidence by Graham-Rowe and colleagues [12] suggested that a number of intervention approaches have the potential to reduce car use, as some of the methodologically strongest studies (controlled trials) demonstrated that car use could be reduced. However the authors were unable to draw firm conclusions, as they did not conduct a meta-analysis, and there were several other limitations of their review. Firstly, the authors did not consider the efficacy of interventions to promote alternatives to car use, but focussed only on car reduction strategies. Secondly, the Graham-Rowe review included all study designs, focusing on the range of evidence rather than on the most robust available. Finally, the review did not consider the specific content of the interventions and was therefore unable to address the questions regarding the active ingredients of the intervention and its mechanisms of action; however it is this level of detail that is likely to be of most use to policy makers interested in promoting behaviour change.

A recent review by Bird and colleagues [13] considered the relation between intervention content (Behaviour Change Techniques) and efficacy of interventions to increase walking and cycling, and found that prompting self-monitoring of behaviour and prompting intention formation could be effective techniques to increase active travel behaviours. However, this review did not address interventions aiming to reduce car journeys. Further, the authors concluded that future research should explore intervention content more extensively and examine, for example, the role of theory.

Interventions informed by psychological theory are hypothesised to show greater efficacy than non-theory based studies. Theory-informed interventions are more likely to target theoretically consistent or empirically supported mechanisms of behaviour change [14]. Interventions may be described as theory-based but the foundation for this is often unclear [15]. It is important that there is examination of the extent to which existing interventions are informed by theory and identification to the theoretical approaches associated with efficacy.

Identifying the circumstances in which different types of interventions are likely to be effective is important for intervention evaluation. Behavioural frameworks, such as the Behaviour Change Wheel aim to do this by categorising the type or types of intervention approach. Context is hypothesised as key to intervention design and implementation, yet it is rarely considered in theory or empirical research.

Accurate descriptions of behavioural interventions are also crucial to understanding intervention efficacy. Minimal intervention detail reporting standards are proposed [16] for outlining characteristics including setting, provider, and format. However, progress with regard to intervention content was limited due to a lack of consensus regarding a shared language for describing interventions. The development of behaviour change taxonomies [17] advanced a common language of intervention content, allowing the identification of active ingredients.

The current systematic review considers the efficacy of behavioural interventions to reduce car use and addresses the limitations in the current literature. The review includes only the most robust study designs and provides a critical appraisal of the methodological quality of the existing evidence. Further, the review considers the specific approach, theoretical basis and content of existing interventions and explores how this relates to efficacy.

### Objectives

The overall objective of this review is to critically evaluate the available evidence of all controlled studies comparing the effects of behavioural interventions to reduce car use for journeys made by adult participants with outcome measures of transport behaviours against no interventions or alternative interventions.

The primary aims of the review are to establish whether current behavioural interventions show efficacy in reducing car use and to explore intervention development and content and how they relate to efficacy.

## Methods

### Protocol and registration

A protocol for this review was registered with the PROSPERO international prospective register of systematic reviews (registration number CRD42011001797) at the Centre for Reviews and Dissemination, University of York, UK (http://www.crd.york.ac.uk/PROSPERO/).

### Eligibility criteria

Inclusion criteria for eligibility of studies to be included in the review were:

i. Participants
  Studies of adults (mean ≥18 years) in the context of their transport journeys. Studies including children were eligible only if parents were targeted by the intervention and outcome assessments.
ii. Interventions
  Studies comprising behavioural or behavioural and structural interventions were included. Behavioural interventions were defined as those which targeted a change in behaviour (for example, providing feedback on travel behaviour). Structural interventions were defined as those which targeted a change in the environment (for example, building a new cycle route). Rationale for the focus on behavioural interventions (alone or in conjunction with structural interventions) was to identify the active ingredients for behaviour change in behavioural interventions. Interventions delivered at an individual level or community level were eligible, but population-level interventions were excluded.
iii. Comparison
  Studies were eligible if they included a control arm receiving no (or minimal) intervention or an alternative intervention.
iv. Outcomes
  Studies were included if they reported a transport-related behavioural outcome assessment for travel taken in the context of repeated journeys, for example travel exclusively for holidays was excluded. As informed by the Graham-Rowe review, these include: number of trips; frequency or proportion of journeys; journey duration; and distance travelled by different modes of transport. This allows the review to deliver evidence on a broad, but relevant, range of outcomes in relation to transport behaviour change.
v. Study design
  Studies were eligible for the review if the study design was: Randomised Controlled Trial, Cluster Randomised Controlled Trial, or Controlled Before and After study. The rationale for this was to include only the most robust designs to assess the efficacy of the interventions, allowing greater confidence in the conclusions.
  Further inclusion criteria specified that studies were available in English and were peer reviewed publications.

### Information sources

Comprehensive searches of the following databases: Applied Social Sciences Index and Abstracts (ASSIA), Ovid Embase, Ovid medline, Ovid PsycInfo, Scopus, Sociological Abstracts, Transportation Research Information Service (TRIS), Transportation Research International Documentation (TRID), and Web of Science were completed by an information specialist librarian in January 2013. No date limit was applied to the searches. Reference cross checking and contact with relevant authors within the field was also utilised.

### Search strategy

An example full electronic search strategy for PsycINFO database, including limits applied is available as an Additional file 1. Full search strategies are available from the first author on request.

### Study selection

Studies were selected for the review in a two-step process following searches and de-duplication. In the first step, titles/abstracts of all studies were checked against the initial inclusion/exclusion criteria by the first author (document available on request). A random sample of all titles/abstracts were also independently screened by a second coder. Disagreements were resolved through discussion, and a third coder was consulted if agreement could not be reached. The full text publications of studies meeting the initial inclusion criteria were accessed. The first author screened full texts against a more detailed inclusion/exclusion criteria (available on request), and excluded studies and reasons for exclusion were documented. A second coder independently screened a sample selected at random. Disagreements were resolved through discussion, and a third coder was consulted if agreement could not be reached. No blinding procedures were used regarding author’s names, institutions, or journal. Studies which were found through alternative means, such as reference cross- checking, were subjected to the same screening process as those which emerged from the searches.

### Data collection process

Data from all papers included in the review were extracted by the first author using a specifically designed data extraction form (available on request). No blinding procedures were used regarding author’s names, institutions, or journal. An independent coder checked a randomly selected sample. Data extraction was informed by the standard extraction strategies set out in the Cochrane Handbook of Systematic Reviews [18]. The data extracted from each study included information on: participants, interventions, comparisons, outcomes, and study design.

Authors of all studies included in the review were contacted and asked to share relevant information regarding the intervention and the results (for example, intervention manuals, and additional data). They were asked to respond within two weeks, if no response was received within two weeks a reminder was sent.

### Data items

Data were extracted from all studies for the following variables (where available):

i. Participants
  Inclusion and exclusion criteria for participation; total number of participants; participant flow through the study (including randomisation and attrition); geographical location (including country, rural vs urban setting); and socio-demographics (including gender, age, income and education).
ii. Interventions

### Intervention characteristics

Davidson and colleagues [16] stressed the importance of considering the minimal detail for reporting interventions (delivery and content) in behavioural medicine, based on the CONSORT reporting guidelines [19] for reporting of Randomised Controlled Trials in medical journals. Following on from these recommendations information on the characteristics of interventions included in this review was extracted and coded. Interventions were distinguished as behavioural or behavioural and structural. Other information was extracted on: intervention provider; mode and format of delivery; intensity and duration of the intervention; and assessment of fidelity of intervention delivery. Information was extracted for intervention and control arms.

### Theoretical basis

Methods of intervention development were coded to understand the theoretical basis of interventions to explore the efficacy of theory-based and non-theory based studies. Interventions were divided into: top-down (developed by researchers, policy makers); bottom-up (developed by input from target populations); or mixed approach. Information was extracted regarding the theoretical underpinnings of the included interventions utilising the Theory Coding Scheme [15] and the revised Theory Domain Framework [20]. The Theory Coding Scheme tool reliably describes the theoretical basis of a behavioural intervention and assesses the extent to which interventions are theory-based [15]. The revised Theory Domain Framework was developed from recognition of the difficulties inherent in identifying successful behaviour change mechanisms. The integrative framework aimed to synthesise the wide range of behaviour change theories. These tools assimilate the theoretical evidence base of interventions.

The Theory Coding Scheme consists of 19 items which are completed for all intervention and control arms for each included study in the review. These items are then summarised into six categories: reference to underpinning theory; targeting of relevant theoretical constructs; using theory to select recipients or tailor interventions; measurement of constructs; testing of mediation effects; and refining theory.

The revised Theory Domain Framework consists of 14 domains and 84 component constructs. The 14 domains were assessed for their presence or absence for each study included in the review. Information was extracted for intervention and control arms. The domains are: knowledge; skills; social/professional role and identity; beliefs about capabilities; optimism; beliefs about consequences; reinforcement; intentions; goals; memory, attention and decision processes; environmental context and resources; social influences; emotions; and behavioural regulation.

### Intervention frameworks

The content of each intervention and control arm was also mapped onto two existing intervention frameworks. These frameworks categorise the type or types of intervention approach. Frameworks can be used to identify the circumstances in which different types of interventions are likely to be effective. The first framework is the Behaviour Change Wheel [21], systematically developed from existing intervention frameworks. The Behaviour Change Wheel targets a broad range of behaviours and is therefore supplemented by the Behavioural Insights Toolkit [22], designed to aid the development and evaluation of real world policies specifically in a transport context.

In this review interventions were categorised in terms of the first two of the three layers of the Behaviour Change Wheel: COM-B (Capability Opportunity Motivation – Behaviour) system and intervention functions. Intervention and control arms were coded in terms of whether they address capability (psychological or physical); opportunity (social or physical); and motivation (reflective or automatic). Included studies were also coded as to whether they targeted each of the nine intervention functions: education; persuasion; incentivisation; coercion; training; enablement; modelling; environmental restructuring; restrictions. Interventions were not coded in relation to the final layer, policy changes, as population level interventions were excluded from this review. Interventions were also categorised in terms of the eight determinants of behaviour in the Behavioural Insights Toolkit: attitudes; emotions, social, cultural and moral norms; structural factors; cost; habit; knowledge and awareness; and capability and self-efficacy.

### Behaviour change technique taxonomy

The content of each intervention was considered using the CALO-RE taxonomy [23]. This 40 item taxonomy was selected as it was specifically developed to assess behaviour change techniques in physical activity interventions. The psychometric properties of this taxonomy have been previously reported [23]. Individual behaviour change techniques present in intervention and control arms are coded for their presence or absence in each intervention arm. The Behaviour Change Taxonomy (v1) [24] was consulted if any techniques were unable to be categorised using the CALO-RE taxonomy. This more comprehensive taxonomy covers a wider range of behaviours and was published subsequent to the review protocol registration. A randomly selected sample was checked by an independent coder for reliability purposes.

iii. Comparisons
  All of the elements of the studies for the control arm(s), including participants, intervention, and outcomes, were extracted.
iv. Outcomes
  Continuous primary outcomes measuring transport behaviours were extracted including: frequency of trips by different modes of transport; proportion of trips by different modes; duration of trip by different modes; and distance travelled. Dichotomous primary outcome measures include: swap to alternative modes of transport.
v. Study design
  Study design was defined using the Cochrane Collaboration handbook [18] as: Randomised Controlled Trial, Cluster Randomised Controlled Trial or Controlled Before and After study.

### Adverse events and compliance

Data on adverse events and assessment of compliance with the intervention were also extracted if present. Definitions of adverse events and compliance were those used by the authors of the included studies.

### Risk of bias in individual studies

The first author extracted information to appraise the methodological quality of the studies included in the review, using the Cochrane Collaboration risk of bias tool [25]. This tool takes into account risk of bias in relation to seven main areas: sequence generation, allocation concealment, blinding participants and personnel, blinding outcome assessors, reporting of incomplete outcome, selective reporting, and other sources of bias. Each area was allocated one of the following judgements: ‘low risk of bias’, ‘high risk of bias’, or ‘unclear risk of bias’. The tool was originally developed for use with Randomised Controlled Trials and was adapted appropriately for Cluster Randomised Controlled Trials and Controlled Before and After studies for the purpose of this review. A randomly selected sample of studies was checked by an independent coder for risk of bias assessments.

### Synthesis of results

#### Meta-analysis

Studies varied in their outcome assessments with some studies measuring a reduction in car use and others assessing an increase in alternative, more active modes of travel. Meta-analyses were therefore performed separately for car reduction and promotion of alternative modes. Studies with sufficient available data, which were able to be meaningfully pooled by outcome measure, were included in the meta-analyses. Random effects models were utilised due to the heterogeneity of interventions included in the review.

#### Narrative synthesis

The results from this review were also integrated in a conceptual narrative following the PRISMA statement (http://www.prisma-statement.org/) [26] guidance. The narrative synthesis incorporates studies which are excluded from the meta-analysis because their main outcome measures cannot be pooled or because there is a lack of available data.

#### Additional analyses

Exploratory analyses consider relations between the presence or absence of specific behaviour change techniques and intervention effect sizes, with the aim of determining which techniques are associated with larger effect sizes to inform future intervention studies.

## Results

### Study selection

**Figure 1:**
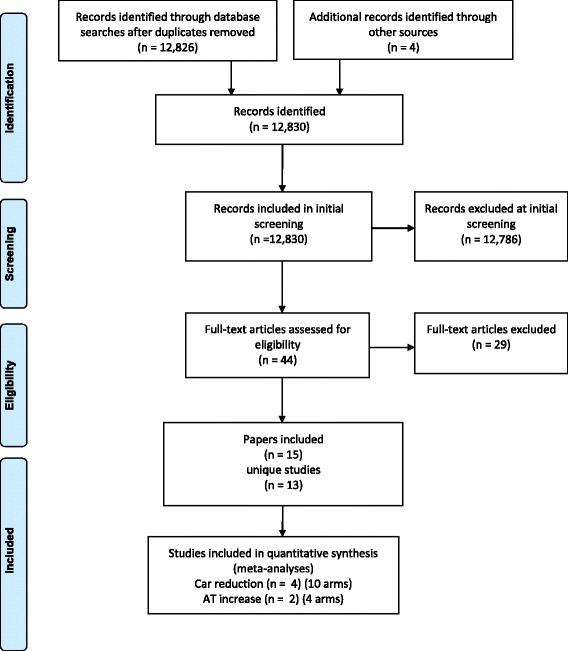
**Flow diagram.** Flow diagram of inclusion of studies in the review (adapted from PRISMA, 2009 [26]).

A total of 12,826 records were identified in the database searches following de-duplication. From the first screening of titles/abstracts, 40 references were identified for second screening. Approximately 20% of all references were randomly selected and checked for eligibility by a second coder at initial screening. An agreement rate of 82% was achieved. Disagreements were resolved through discussion or through consultation with a third coder. Four papers were identified from reference cross-checking and contacting relevant authors in the field. Full text of these 44 papers were sought. Following second screening, 15 papers (representing 13 unique studies) met the eligibility criteria for inclusion in the review. A random 25% of all full texts were checked for eligibility at secondary screening with an agreement rate of 89% (kappa 0.77). Disagreements were resolved through discussion or through consultation with a third coder. See Figure 1 for more details.

### Study characteristics

Table 1 shows a summary of the characteristics of the included studies. This section addresses the types of study designs employed and the nature of comparison arms in included studies. The methodological quality of the studies is assessed in a separate section.

**Table 1:** **Summary of included study characteristics**

	**Participants** ^**1**^	**Participants** ^**2**^	**Intervention**	**Comparator intervention**	**Primary outcomes** ^**4**^	**Study design**
Aittasalo 2012 [27]	241	Intervention: 71% female, 44 years	Behavioural	None or minimal	Duration	RCT
		Control: 66% female, 45 years			Non-car	
Armitage 2011 [28]	701	56% Female	Behavioural	None or minimal	Frequency car	RCT
		20-64 years				
Bamberg 2006 [29]	241	47% Female	Behavioural & Structural	None or minimal	Proportion	RCT
		Mean 29 years			Car and Non-car	
Bamberg 2013 [30]		72% Female	Behavioural	None or minimal	Proportion	RCT
		Mean 36 years			Car and Non-car	
Ben-Elia 2011 [31]	341	Intervention:	Behavioural	Alternative	Proportion	CBA
		26% Female mean 45 years			Non-car	
		Alternative:				
		40% Female mean 41 years				
Eriksson 2008 [32]	184^3^	Intervention:	Behavioural	None or minimal	Frequency car	RCT
		52% Female mean 53 years				
		Control:				
		46% Female mean 53 years				
Fujii 2005 [33]	292	Unknown	Behavioural	Alternative	Frequency car	CBA
Garvill 2003 [34]	372^3^	51% Female	Behavioural	None or minimal	Frequency car	CRCT
		Mean 44 years				
Jakobsson 2002 [35]	182^3^	Mean ages 42–51 years	Behavioural	None or minimal	Frequency and Distance car	CRCT
Matthies 2006 [36]	578	38% Female	Behavioural	None or minimal	Try-out	RCT
		Mean 45 years			Non-car	
Mutrie 2002 [37]	295	64% Female	Behavioural	None or minimal	Duration	RCT
		Mean 38 years			Non-car	
Tertoolen 1998 [38]	350	Unknown	Behavioural	None or minimal	Distance car	RCT
Thøgersen 2008 [39]	1071	Unknown	Behavioural	Alternative	Frequency Non-car	CBA

^1^Number of participants at randomisation.

^2^Participant characteristics.

^3^Estimated based on number of households, assuming 2 adults per household participated.

^4^All continuous measures except for the Matthies Try-out assessment which is dichotomous.

- Participants:
  The review represents a total of 4895 participants (mean 377 per study, SD = 261.9, range 140 to 1071). The percentage of females in the studies ranged from 35% to 72%. Age range was 18 to 69 years (mean age range 28.6 to 45 years, where reported). Socio-economic status was variable (where reported) but mostly medium to high. Participants were largely urban dwellers in European countries.
- Interventions

### Intervention characteristics

All studies were behavioural only interventions, with the exception of the Bamberg [29] study which also included structural components. There were 27 intervention arms across the 13 studies; six studies included one intervention arm [27-29,32,34,37], three included two intervention arms [30,31,33], two included three intervention arms [35,36], one study included four [38], and one study five [39]. All interventions were delivered at the level of the individual or household, with the exception of the Aittasalo study which also included some delivery in a group setting. In the majority of intervention arms the intervention was delivered by members of a research team (alone or in combination with others); with the exception of the intervention arms in the Bamberg [29], Bamberg [30], Garvill et al., and Thøgersen studies where the intervention was delivered by others, for example a local transport company. In seven intervention arms across five studies [28,30,33,37,38] the intervention was delivered through written materials; in six intervention arms across two studies [35,38] the intervention was delivered in face to face meetings; 12 arms of six studies [27,30,32,33,36,39] delivered the intervention using some combination of written self-help materials, face to face meetings, calls and/or emails. One arm of the Ben-Elia study delivered the intervention online and the other arm of this study received an intervention via a combination of smartphone and online. Twenty four intervention arms in eleven studies were delivered to participants at home; one intervention arm [37] was delivered at work; one intervention arm [27] was delivered at a combination of home and work; and one intervention arm in the Ben-Elia study was delivered at both home and in the car. The duration of the active period of intervention varied from 1 week to 24 weeks (Mean = 7.07, SD = 6.41). The frequency of contact ranged from two contacts per week to one contact every six months of active intervention. The majority of studies involved contacts with participants on average between once per week and once per month during the active intervention period. The intervention target was the intervention recipient in all cases. Only one study [27] included a fidelity check to assess whether the intervention was delivered as intended.

Details of all of the intervention arm characteristics can be found in Table 2.

**Table 2:** **Summary of included study intervention arm components**

	**Arm name**	**Setting**	**Provider**	**Format**	**Duration (weeks)**	**Frequency intensity of active intervention**
Aittasalo 2012 [27] Intervention	STEP	Individual + Group	Researcher + Other	Face to face + written self help + emails	24	1 contact per 2 weeks
Armitage 2011 [28] Intervention	Implementation Intention	Individual	Researcher	Written self help	4	1 contact per 4 weeks
Bamberg 2006 [29] Intervention	Intervention	Individual	Other	Written self help	6	1 contact per 6 weeks
Bamberg 2013 [30] Intervention	Dialog	Individual	Other	Written self help + calls	2	2 contacts per week
Bamberg 2013 [30] Alternative	Standardised	Individual	Other	Written self help	1	1 contact per week
Ben-Elia 2011 [31] Intervention	Yeti	Individual	Researcher	Online	10	1 contact per week
Ben-Elia 2011 [31] Alternative	Monetary	Individual	Researcher	Online	11	1 contact per week
Eriksson 2008 [32] Intervention	Intervention	Individual	Researcher	Face to face + written self help	1	1 contact per week
Fujii 2005 [33] Intervention	Plan	Individual	Researcher	Written self help	1	1 contact per week
Fujii 2005 [33] Alternative	Advice	Individual	Researcher	Written self help	1	1 contact per week
Garvill 2003 [34] Intervention	Intervention	Household	Other	Face to face + written self help + calls	1	2 contacts per week
Jakobsson 2002 [35] Intervention	Charge	Household	Researcher	Face to face	2	1 contact per week
Jakobsson 2002 [35] Alternative 1	Charge + plan	Household	Researcher	Face to face	2	1 contact per week
Jakobsson 2002 [35] Alternative 2	Extend charge + plan	Household	Researcher	Face to face	4	1 contact per 2 weeks
Matthies 2006 [36] Intervention	Commitment + free ticket	Individual	Researcher	Face to face + written self help	4	1 contact per week
Matthies 2006 [36] Alternative 1	Free ticket	Individual	Researcher	Face to face + written self help	4	1 contact per week
Matthies 2006 [36] Alternative 2	Commitment	Individual	Researcher	Face to face + written self help	4	1 contact per week
Mutrie 2002 [37] Intervention	Intervention	Individual	Researcher	Written self help	24	1 contact per 24 weeks
Tertoolen 1998 [38] Intervention	Environmental information	Individual	Researcher	Face to face	8	1 contact per 2 weeks
Tertoolen 1998 [38] Alternative 1	Cost information	Individual	Researcher	Face to face	8	1 contact per 2 weeks
Tertoolen 1998 [38] Alternative 2	Environment + Cost information	Individual	Researcher	Face to face	8	1 contact per 2 weeks
Tertoolen 1998 [38] Alternative 3	No information	Individual	Researcher	Face to face	1	1 contact per week
Thøgersen 2008 Intervention [39]	Free travelcard	Individual	Other	Written self help + calls	12	1 contact per 8 weeks
Thøgersen 2008 Alternative 1 [39]	Customised Timetable	Individual	Other	Written self help + calls	12	1 contact per 8 weeks
Thøgersen 2008 Alternative 2 [39]	Free travelcard + customised timetable	Individual	Other	Written self help + calls	12	1 contact per 8 weeks
Thøgersen 2008 Alternative 3 [39]	Plan	Individual	Other	Written self help + calls	12	1 contact per 8 weeks
Thøgersen 2008 Alternative 4 [39]	Free travelcard + plan	Individual	Other	Written self help + calls	12	1 contact per 8 weeks

### Theoretical basis

All studies included were top-down in nature; none report any input of target populations in the development of the intervention. The theoretical underpinnings of each study were further considered using the Theory Coding Scheme and the Theory Domain Framework.

**Figure 2:**
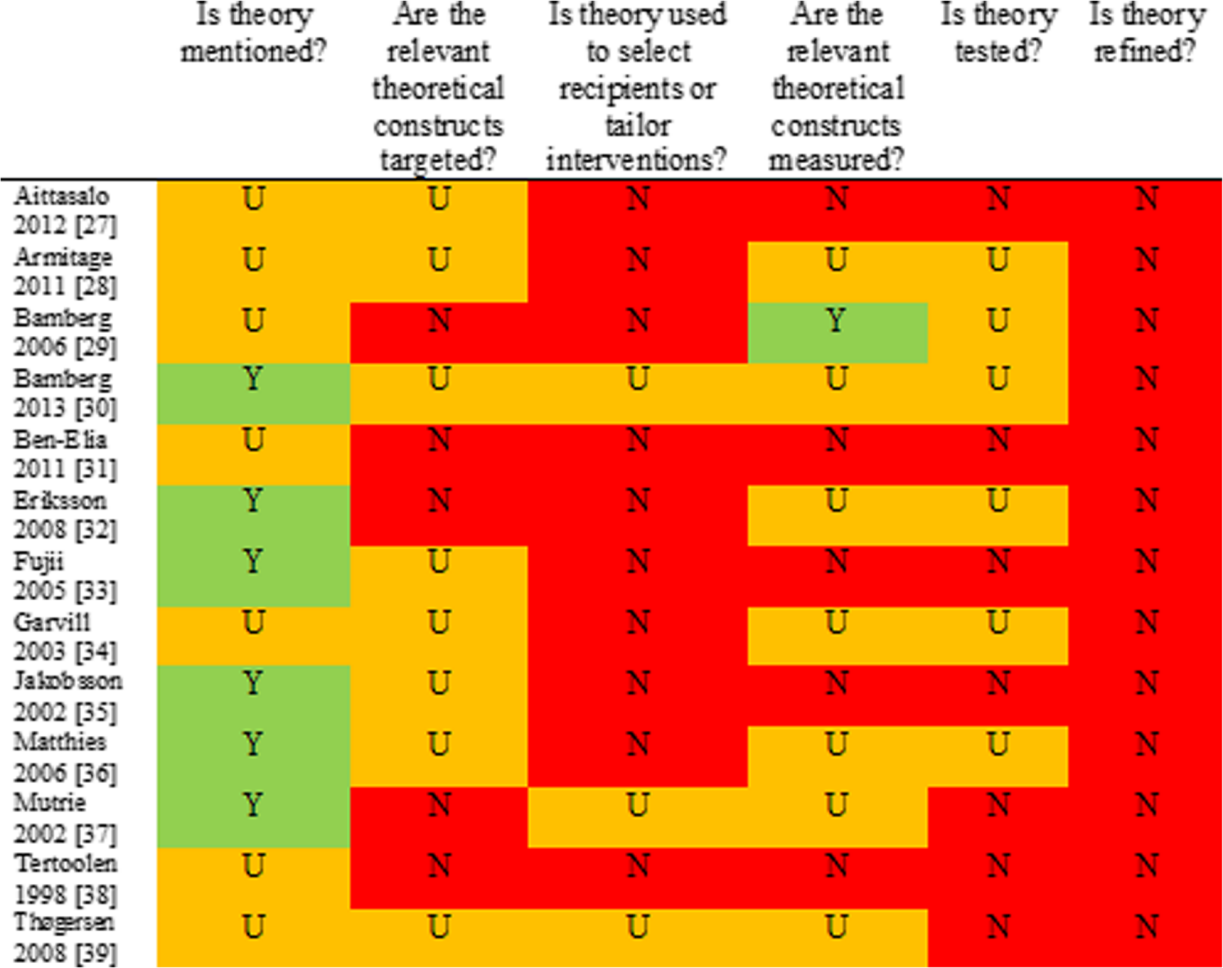
**Summary item scores for included studies on the Theory Coding Scheme.**

Figure 2 shows the results for individual studies in the review in relation to the 6 summary questions of the Theory Coding Scheme. Six studies were informed by theory [30,32,33,35-37], for the remaining interventions the theoretical basis was less clear. The most commonly mentioned theories were social cognition theories [e.g., Theory of Planned Behaviour [40]], habit theory [41], or a mixture of both. However, although some interventions were informed by theory the expected theoretical constructs were not always targeted or measured. Further, few studies selected participants or tailored the intervention based on theory. Theory-testing was poor and behavioural models were not refined on the basis of adequate analyses.

Table 3 shows the results for the intervention arm(s) of individual studies in relation to the Theory Domain Framework. The results show that a wide range of theoretical domains were present in the studies. Interventions commonly targeted knowledge, goals and behavioural regulation. No studies targeted role identity, optimism, emotion or memory, attention and decision processes.

**Table 3:** **Presence of Theory Domain Framework Categories in included studies**

	**Knowledge**	**Skills**	**Role identity**	**Capabilities**	**Optimism**	**Consequences**	**Reinforcement**	**Intentions**	**Goals**	**Memory, attention**	**Environment**	**Social**	**Emotion**	**Regulation**
Aittasalo 2012 [27]	X			X					X					X
Armitage 2011 [28]									X					X
Bamberg 2006 [29]	X						X				X			
Bamberg 2013 [30]	X					X	X	X	X			X		X
Ben-Elia 2011 [31]	X						X							X
Eriksson 2008 [32]	X	X							X					X
Fujii 2005 [33]	X					X	X		X			X		X
Garvill 2003 [34]									X					X
Jakobsson 2002 [35]							X		X					X
Matthies 2006 [36]	X					X	X	X	X					X
Mutrie 2002 [37]	X								X		X	X		X
Tertoolen 1998 [38]	X								X					X
Thøgersen 2008 [39]	X						X		X					X

Presence of a Theory Domain Framework category in an intervention arm is indicated with an X.

### Intervention frameworks

Table 4 shows the mapping of the intervention arm(s) of individual studies in the review on to the Capability Opportunity Motivation Behaviour System (COM-B) of the Behaviour Change Wheel. Most interventions were categorised as targeting Psychological Capability or Reflective Motivation. Very few interventions targeted either Social or Physical Opportunity. None addressed Physical Capability.

**Table 4:** **Presence of components of the Capability Opportunity Motivation - Behaviour (COM-B) system in included studies**

	**Capability-physical**	**Capability-psychological**	**Opportunity-social**	**Opportunity-physical**	**Motivation-reflective**	**Motivation-automatic**
Aittasalo 2012 [27]		X			X	
Armitage 2011 [28]		X			X	
Bamberg 2006 [29]		X		X		X
Bamberg 2013 [30]		X			X	X
Ben-Elia 2011 [31]		X			X	
Eriksson 2008 [32]		X			X	
Fujii 2005 [33]		X	X		X	X
Garvill 2003 [34]		X			X	
Jakobsson 2002 [35]		X			X	X
Matthies 2006 [36]		X			X	X
Mutrie 2002 [37]		X			X	
Tertoolen 1998 [38]		X			X	
Thøgersen 2008 [39]		X			X	X

Presence of a COM-B component in an intervention arm is indicated with an X.

Table 5 shows the mapping of intervention arms of individual studies on to the intervention functions of the Behaviour Change Wheel. Most studies addressed Enablement, Education or Incentivisation. No studies addressed Training, Modelling or Restriction.

**Table 5:** **Presence of intervention functions of the behaviour change wheel in included studies**

	**Education**	**Persuasion**	**Incentives**	**Coercion**	**Training**	**Enablement**	**Modelling**	**Environmental restructuring**	**Restriction**
Aittasalo 2012 [27]	X					X			
Armitage 2011 [28]						X			
Bamberg 2006 [29]	X		X					X	
Bamberg 2013 [30]	X	X	X			X			
Ben-Elia 2011 [31]	X		X			X			
Eriksson 2008 [32]	X					X			
Fujii 2005 [33]	X		X			X			
Garvill 2003 [34]						X			
Jakobsson 2002 [35]				X		X			
Matthies 2006 [36]	X	X	X			X			
Mutrie 2002 [37]	X		X			X			
Tertoolen 1998 [38]	X	X				X			
Thøgersen 2008 [39]	X		X			X			

Presence of a Behaviour Change Wheel Intervention Function in an intervention arm is indicated with an X.

Table 6 shows the mapping of intervention arms in the studies included in the review on to the Behavioural Insights Toolkit. Studies were largely categorised as targeting Habit, Knowledge and awareness, or Costs. There were no studies targeting Emotions.

**Table 6:** **Presence of components of the Behavioural Insights Toolkit in included studies**

	**Attitudes**	**Emotions**	**Norms**	**Structural**	**Costs**	**Habit**	**Knowledge/awareness**	**Capability/self-efficacy**
Aittasalo 2012 [27]	X					X	X	X
Armitage 2011 [28]						X		
Bamberg 2006 [29]				X	X	X	X	
Bamberg 2013 [30]	X		X		X	X	X	
Ben-Elia 2011 [31]					X	X	X	
Eriksson 2008 [32]						X	X	
Fujii 2005 [33]			X			X	X	
Garvill 2003 [34]						X	X	
Jakobsson 2002 [35]					X	X	X	
Matthies 2006 [36]					X	X	X	
Mutrie 2002 [37]	X						X	X
Tertoolen 1998 [38]						X	X	
Thøgersen 2008 [39]					X	X	X	

Presence of a Behavioural Insights Toolkit component in an intervention arm is indicated with an X.

### Behaviour change technique taxonomy

A total of 20 existing behaviour change techniques were used in the intervention arms, as identified from the CALO-RE taxomomy. The most common Behaviour Change Techniques were: ‘action planning’ (n = 7); ‘prompt self-monitoring of behaviour’ (n = 16); ‘provide information on where and when to perform the behaviour’ (n = 16); and ‘provide information on how to perform the behaviour’ (n = 11). There were also 8 additional behaviour change techniques identified, which could not be coded from the CALO-RE taxonomy, and for these the V1 taxonomy was consulted. One was coded as ‘material incentive (behaviour), one as ‘pros and cons’, one as ‘one as ‘verbal persuasion of capability’, and one as ‘punishment’. One additional BCT could not be coded within the CALO-RE or V1 taxonomy and this was described as ‘prompt consideration of contextual constraints’. This technique may be specifically relevant to travel behaviours. The mean number of Behaviour Change Techniques present in an intervention arm was 4.59 (SD = 3.17, range 1 to 15).

c) Comparators
  All studies in the review compared one or more experimental intervention arms to a no or minimal control arm, with the exception of the three studies [31,33,39] which included a comparison arm receiving an alternative intervention of similar intensity, as described in the inclusion criteria. The intervention arm is identified in all studies by selecting the most intensive intervention in terms of behaviour change techniques delivered or where the intervention is highlighted in the paper as innovative and is compared to a standard, existing intervention.
d) Outcomes
  A wide range of transport behaviour assessment outcomes were apparent in the included studies: frequency of trips (n = 10) – nine relating to car trips [28,32-35] and one to non-car trips [39]; proportion of trips (n = 5) – two relating to car trips [29,30] and three to non-car trips [29-31]; trip duration (n = 3) – all relating to non-car trips [27,37]; trip distance (n = 2) – all relating to car trips [35,38]; and mode swap (n = 1) from car to no-car [36]. All of the main outcome measures were self-reported, although in one study [31] there is some objective verification, in the form of a transponder fitted to the car which indicated when a journey was made, although the results still rely on self-reports. None of the studies provide any evidence for the reliability or validity of the primary outcome measures.
e) Study designs
  There were eight Randomised Controlled Trials [27-30,32,36-38]; two Cluster Randomised Controlled Trials [34,35], and three Controlled Before and After studies [31,33,39] in the review.

### Compliance and adverse events

Compliance of participants with the intervention was only assessed in two studies [27,28]. In the Armitage study there were low rates of compliance (25%) but in the Aittasalo study rates were higher (37% to 80% for different parts of the intervention at different time points). Only one study [27] reported on the presence of any adverse events: between 8% and 15% in the intervention arm and between 14% and 17% in the control arm, although the exact nature of the events was not specified.

### Risk of bias within and across studies

**Figure 3:**
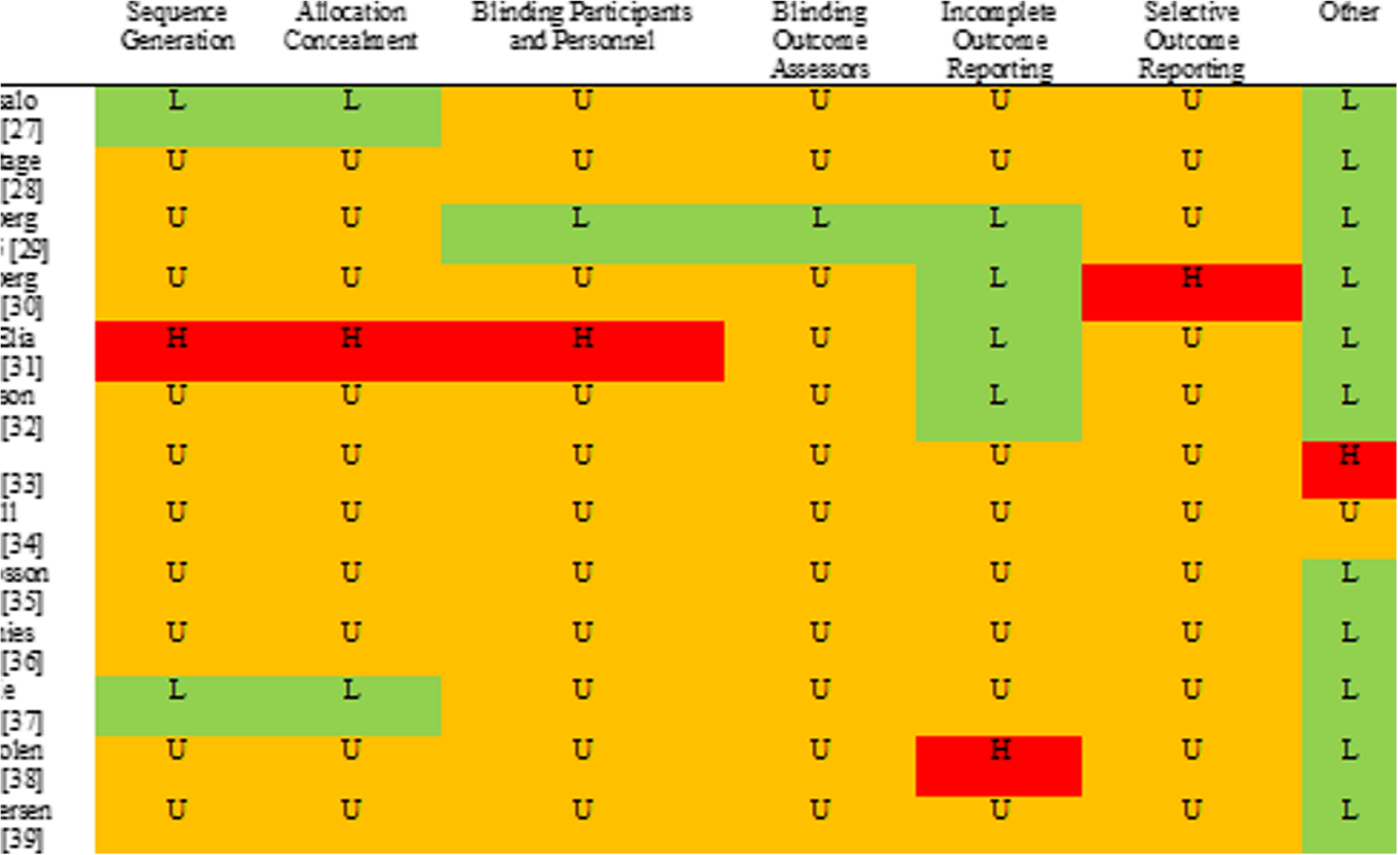
**Methodological quality of included studies** ^**1**^ **.**

Figure 3 shows the methodological quality assessments for the studies included in the review.

Only two studies described the randomisation sequence generation in sufficient detail or adequately described satisfactory attempts to conceal allocation [27,37]. One study reported acceptable blinding procedures for participants and intervention providers [29] and only one reported acceptable procedures for blinding of outcome assessors [30]. Other studies did not provide enough information to judge whether the blinding procedures were acceptable. Four of the included studies described the sample attrition adequately [29-32] but none give full reasons for attrition. Concerns regarding selective reporting bias were raised due to a lack of published protocols, and the absence of sufficient data for a number of studies. Other sources of potential bias were also considered including the unit of analysis where there is clustering of participants and pre-existing baseline differences in Controlled Before and After studies. None of the Controlled Before and After studies reported appropriate steps to avoid contamination between conditions. Only one study explicitly reported intention-to-treat analysis [37]. Overall, the methodological quality of included studies is unclear due to lack of reporting of relevant information.

Risk of bias across the review is appraised as considerable due to the unclear methodological quality of the individual included studies. Assessments suggested that selection bias is present in almost all studies, and that for the majority of studies there is insufficient evidence of blinding of participants, of those delivering the intervention, or of outcome assessors. Since the outcome measures are largely self-report there is a considerable chance that the results may have been affected by bias, although the difficulty associated with blinding participants in behavioural interventions is acknowledged.

### Synthesis of results

#### Meta-analyses of outcome by efficacy

Studies are grouped together using the intervention target (reducing car use or increasing more active travel modes) and the outcome measure (frequency, proportion of trips, duration, distance) so that pooled results are easily comparable. With respect to car reduction, there was only sufficient data relating to the frequency of car journeys to perform a meta-analysis. With respect to increasing more active travel modes, only the proportion of journeys by more active travel modes had sufficient data. The reduction of car frequency meta-analysis is presented below. The meta-analysis examining the promotion of more active travel modes is presented in Additional file 2.

### Car reduction: frequency of trips

Four studies [28,32,33,35] were eligible for inclusion in the meta-analysis of car trip frequency outcomes, with 10 intervention arm outcomes available for the analysis.

**Figure 4:**
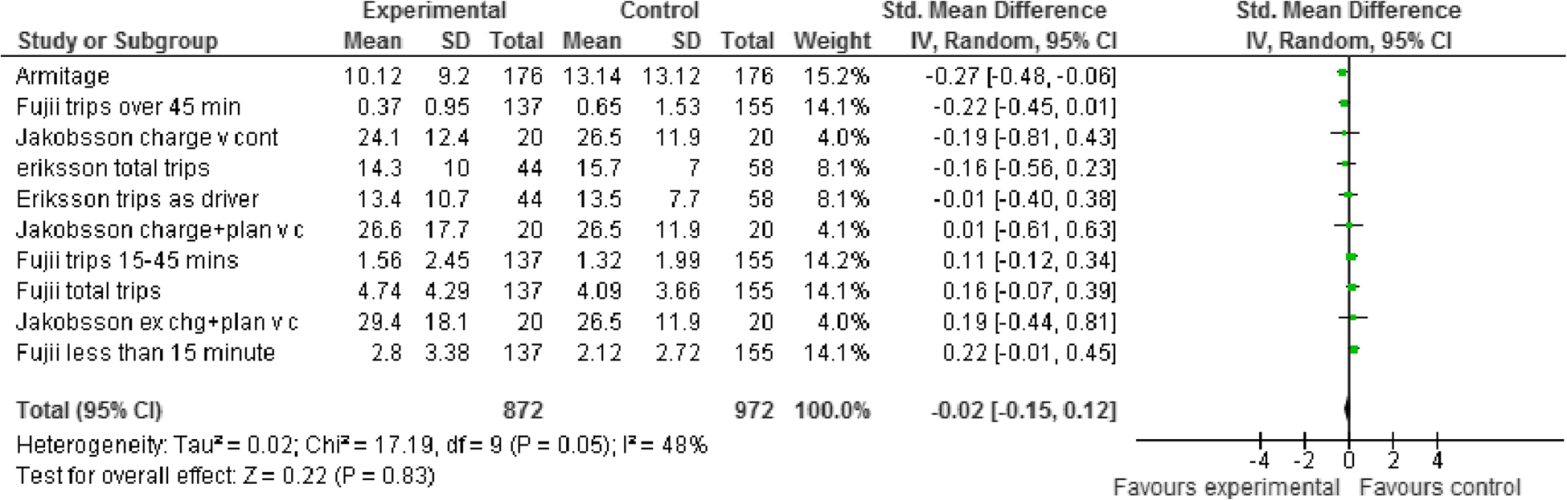
**Behavioural interventions to reduce car use.**

Figure 4 shows the forest plot of the comparison intervention arms and controls in reducing car use using a random effects model. Results show that interventions have no significant effect with a standardised mean difference (SMD) of −0.02 (95% CI = −0.15, 0.12) with moderate heterogeneity (*I*^*2*^ *= 48% and* chi^2^ = 17.19 [df = 9, p = 0.05]). Potential for publication bias was explored by plotting the inverse of the standard errors of effect estimates using a ‘funnel plot’. This plot was assessed visually to explore symmetry. No evidence of asymmetry was present. The evidence suggests there is no efficacy of the behavioural interventions in these studies to reduce car use frequency.

### Narrative synthesis

The primary objective of this synthesis is to consider the efficacy of the studies excluded from the meta-analyses. To assess efficacy effect sizes (Cohen’s *d*) are calculated using the last assessment of the outcome of interest post-intervention, where the effect size can be calculated from data in the paper or on request from authors. Where no effect size can be calculated the conclusions of the publication are presented.

The studies are presented by type of outcome and sub-divided into those studies which targeted car use reduction and those which aimed to promote more active modes of transport. Studies targeting car reduction outcomes are included below. Studies relating to more active travel promotion outcomes are synthesised in Additional file 3.

i. Frequency
  There were 5 studies [28,32-35] which aimed to reduce car use and for which frequency of car trips was the main outcome. Four of these studies are included in the meta-analysis [28,32,33,35]; all are discussed below.

#### Studies included in meta-analysis

In the study by Armitage [28] participants in the intervention arm received a request to form an implementation intention to reduce single occupancy car use. There was no-significant effect (*d* = −0.27); although the effect size was larger (*d* = −0.34) among those who complied with the intervention instructions and actually formed an implementation intention. It is important to highlight that both the intervention and the control arm participants actually *increased* their single occupancy car use during the intervention, but study authors argue that the intervention served to limit the extent of the expected seasonal increase in car use in the intervention arm.

In the Eriksson study [32] intervention arm participants were asked to complete a prospective car diary during a home visit. They were asked to decide if they would use the car for each trip and to provide justifications for their choices. They were also asked to consider reduction of spontaneous car trips. Participants in the intervention arm were also provided with a list of car reduction strategies. This study showed no effect for reducing frequency of car trips as driver (*d* = −0.01) and for reducing frequency of total car journeys (*d* = −0.16).

In the Fujii study [33], parents of school children were allocated to one of two alternative intervention arms: a planning arm and a standard advice arm. In the planning condition participants were asked to make plans to reduce their car use. In the advice arm participants received a diagnostic checklist with figures and comments informing them how they could reduce their car use. In this study the total frequency of car journeys showed a non-significant *increase* as a result of the intervention (*d* = 0.16), therefore in the opposite direction to the aim of the intervention. The frequency of car journeys of less than 15 minutes duration and from 15–45 minutes duration showed similar patterns of *increase* (*d* = 0.22 and *d* = 0.11 respectively). However, there were pre-existing baseline differences in this Controlled Before and After study. Before the intervention, participants in the control made fewer car journeys but this increased during the period of the intervention. In comparison, the intervention participants made more car trips at baseline but decreased use during the intervention. These pre-existing differences mean that the results must be interpreted with caution, particularly for total frequency. In relation to frequency of car journeys >45 minutes duration, there was a non-significant *reduction* (*d* = −0.22). Again there were baseline differences between the two arms, with control participants making fewer long trips at baseline but increasing during the period of the intervention and the intervention participants making a greater number of longer car journeys at baseline but decreasing during the intervention period. The effects in this study may be underestimated and therefore the results should be interpreted with caution. Further, the effects may have been underestimated since the comparison was made with an alternative intervention, which may include some active ingredients, for example self-monitoring, rather than a no or minimal intervention control.

In the Jakobsson study [35], there were 3 intervention conditions: a Charge condition (participants were charged for 2 weeks for using their cars above and beyond baseline rates of car use); a Charge plus planning (participants were charged for using their car above baseline rates for 2 weeks and engaged in a planning activity to avoid car use); and an Extended charge plus planning intervention (charged for 4 weeks for usage above baseline rates and engaged in a planning activity). The fourth arm was a no intervention control. Compared to a no intervention control the Charge intervention showed a non-significant effect on car use reduction (*d* = −0.19). The Charge plus planning also showed no significant effect (*d* = 0.01) on car use. The Extended charge plus planning intervention, showed a non-significant *increase* in car journey frequency (*d* = 0.19). Due to pre-existing baseline differences, with the control arm participants making fewer journeys at baseline compared to both of the planning arms, the results from this study must be interpreted with some caution.

#### Studies not included in the meta-analysis

There was only one study [34] relating to reducing car use which was not eligible for inclusion in the meta-analysis, due to a lack of available data. In this study during the intervention period the intervention and control arm participants received slightly different travel diaries to complete. Both travel diaries included a planning section in which participants were encouraged to plan how they would travel for certain trips in the forthcoming week. The intervention diaries differed by prompting the participant to think about the specific contextual conditions of the trip which may affect their decision, for example whether they needed to transport heavy bags. The aim of these questions was to encourage more conscious travel choices by increasing awareness of journey context. In the paper the authors sub-divided the data of those in the intervention and control arms by car habit strength (weak vs strong) on two different measures of habit: response frequency measure [42] and self-reported past frequency of car use. The authors concluded in the paper that the estimated marginal mean (*m* =10.23) for the intervention arm was smaller than the estimated marginal mean (*m* =13.01) for the control arm for car use frequency and that this was a significant difference (p < 0.05).

ii. Proportion
  There were 2 studies [29,30] aiming to reduce car journeys which used proportion of trips as the main outcome. In the Bamberg study [29] those relocating to another city were the target of the intervention. Soon after relocation, the intervention arm received an intervention pack from a local transport company containing a free one day public transport pass and a map of public transport services, timetables, and other related information. The Bamberg [29] study showed a non-significant trend for reducing the proportion of car journeys (*d* = −0.24). However, this outcome was assessed via a limited one-day trip diary, so the results must be interpreted with caution. Other studies in the review have collected outcome data over longer durations. Data collection over shorter periods may be limited by daily fluctuations in travel patterns. Further, there were pre-existing baseline differences between intervention and control participants in this study; with controls making a slightly greater proportion of trips by car at baseline. The results should therefore be interpreted with some caution.
  In the Bamberg [30] study there were 2 intervention arms compared with a no intervention control. The intervention conditions were: a) the dialog condition receiving a tailored intervention package and b) the standard condition receiving what is described as a standardised travel information package, consisting of brochures and leaflets relating to public transport services, active travel routes, and car sharing facilities. The Bamberg [30] study suggested a medium (*d* = −0.54) decrease in car use as a consequence of the main dialog intervention and a small (*d* = −0.17) decrease in response to the alternative standardised intervention. Baseline data was not available for this study, so the results should be interpreted with some caution as it is possible that there were pre-existing baseline differences.
  The evidence relating to decreasing the proportion of car trips is limited and must be interpreted with some caution, and the findings are heterogeneous.

#### Studies included in the meta-analysis

The Bamberg [29], described above, showed a medium effect size (*d* = 0.47) for an increase in the proportion of trips by public transport. However, caution must be applied as this outcome is assessed via a limited one-day trip diary.

In the Ben-Elia study [31], participants were allocated to one of two alternative interventions: a monetary reward or a Yeti smartphone credit reward. The Yeti smartphone credit condition participants also received traffic information delivered directly to their smartphone. In this study there was no significant post-intervention difference (*d* = 0.00) in the proportion of non-car journeys in rush hour (this covered not travelling, or travelling by alternative modes). However, there were pre-existing differences between the intervention and control participants at baseline, with the Monetary intervention participants making a higher proportion of non-car journeys prior to the intervention and reducing this proportion by the follow up assessment, and the Yeti participants making a smaller proportion but increasing by the follow up. Therefore the results must be interpreted with some caution, as it is possible that the effect of the Yeti intervention is underestimated. Further, the effect may be underestimated through comparison with an alternative intervention rather than just a no or minimal intervention control.

#### Studies not included in the meta-analysis

In the Bamberg study [30], described above, there was evidence of a medium post-intervention increase in the proportion of public transport journeys (*d* = 0.41) as a result of the dialog intervention but no effect as a result of the standard intervention (*d* = −0.06). There was no effect on walking or cycling journey proportions in response to the dialog intervention (*d* = −0.05 and *d* = −0.03 respectively) or the standard intervention (*d* = −0.05 and *d* = −0.0 respectively). There is no baseline data available for this study so some caution must be applied.

The results suggest that the evidence relating to increasing the proportion of more active travel journeys is limited, shows high heterogeneity, and should be interpreted with caution.

iii. Duration
  There were no studies with duration of car trips as the main outcome in this review, and therefore there is no evidence relating to this question.
iv. Distance of trips
  In the Tertoolen study [38] there were 5 arms. The first consisted of participants receiving information on the environmental effects of car use and self-monitoring their travel behaviours (Environmental Information condition); the second consisted of participants receiving information on the financial effects (costs) of car use and self-monitoring their travel behaviours (Costs Information condition); the third consisted of participants receiving information on both environmental and financial effects of car use and self-monitoring their travel behaviours (Environmental and Costs Information condition); the fourth consisted of participants receiving no information but engaging in self-monitoring of travel behaviours (No Information condition); and finally, participants receiving no information and not engaging in self-monitoring (Control condition). There was insufficient data to calculate effect sizes for the Tertoolen study; however the study author concluded that there were no effects of the interventions on car use, after controlling for covariates. The evidence relating to efficacy of existing behavioural interventions to decrease car trip distance is weak and inconclusive.
  Overall, the results from the narrative review conclude that the evidence relating to car reduction and increase of more active travel alternatives is limited, inconclusive, and heterogeneous.

### Additional analyses

#### Exploration of BCTs in relation to efficacy

The evidence relating to the efficacy of behavioural interventions to reducing car use and increasing more active travel is highly heterogeneous, therefore further exploratory analyses were conducted to examine aspects of interventions which may be related to efficacy. The association between the presence of behaviour change techniques and intervention effect size was explored. These analyses were able to incorporate some studies relating to alternative outcomes, such as distance and duration, which may be important for health outcomes but were not able to be included into the meta-analyses. These exploratory analyses can be used to inform future interventions, but some caution must be applied since this technique is tentative.

Tables 7 and 8 show the association between the effect sizes of interventions to decrease car use and increase more active travel modes respectively and the presence of individual behaviour change techniques. Studies for which an effect size could be calculated from the available data are included. Studies are ranked from left to right in terms of effect size. Studies which show an effect in the unexpected direction (an *increase* in car use or a *decrease* in active travel) are shaded in grey. All behaviour change techniques present in included studies are listed in the table. The presence of a technique is indicated with an X. Techniques which are not present in any of the included studies are not listed in the tables.

**Table 7:** **Behaviour change technique content coding of car reduction studies by effect size**

**Study name**	**Bamberg 2013 [** 30 **]**	**Armitage 2011 [** 28 **]**	**Bamberg 2006 [** 29 **]**	**Fujii 2005 [** 33 **]**	**Jakobsson 2002 [** 35 **]**	**Bamberg 2013 [** 30 **]**	**Eriksson 2008 [** 32 **]**	**Eriksson 2008 [** 32 **]**	**Jakobsson 2002 [** 35 **]**	**Fujii 2005 [** 33 **]**	**Fujii 2005 [** 33 **]**	**Jakobsson 2002 [** 35 **]**	**Fujii 2005 [** 33 **]**
Intervention arm name	Dialog	Implementation Intention	Intervention	Plan	Charge	Standardised	Intervention	Intervention	Charge + plan	Plan	Plan	Extend charge + plan	Plan
Outcome	Car use (summary measure)	Single occupancy car use (frequency)	Car Use (Proportion)	Car trips >45 mins duration (frequency)	Car use (frequency)	Car use (summary measure)	Total car trips (frequency)	Car trips as driver (frequency)	Car use (frequency)	Car trips 15-45 mins (frequency)	Total car trips (frequency)	Car use (frequency)	Car trips <15mins (frequency)
Effect size^1^	−0.54	−0.27	−0.24	−0.22	−0.19	−0.17	−0.16	−0.01	0.01	0.11	0.16	0.19	0.22
**CALO-RE BCTs** ^**2**^													
1	X			X						X	X		X
2					X				X			X	
5	X												
7	X	X		X			X	X	X	X	X	X	X
8	X												
12	X												
13					X				X			X	
15	X						X	X					
16					X				X			X	
20	X		X			X	X	X					
21	X		X			X	X	X					
28	X												
40	X												
**V1 BCTs**													
Verbal persuasion	X												
Pros and Cons	X												
Incentive			X										
Punishment					X				X			X	
**Additional BCTs**													
Contextual Constraints	X												
**Structural interventions**													
Relocation			X										

Presence of a Behaviour Change Technique in an intervention arm is indicated by an X.

^1^Effect size ranked from most effective (greatest reduction in car use) through to least effective (increases in car use), where effect size could be calculated.

^2^1: Provide information on consequences of behaviour in general; 2: Provide information on consequences of behaviour to the individual; 5: Goal setting (behaviour); 7: Action planning; 8: Barrier identification/problem solving, 12: Prompt rewards contingent on effort or progress towards behaviour; 13: Provide rewards contingent on successful behaviour; 15: Prompting generalisation of a target behaviour; 16: Prompt self-monitoring of behaviour, 20: Provide information on where and when to perform the behaviour; 21: Provide instruction on how to perform the behaviour; 28: Facilitate social comparison; 40: Stimulate anticipation of future rewards.

**Table 8:** **Behaviour change technique content coding of more active travel promotion studies by effect size**

**Study name**	**Bamberg 2006 [** 29 **]**	**Bamberg 2013 [** 30 **]**	**Aittasalo 2012 [** 27 **]**	**Bamberg 2013 [** 30 **]**	**Bamberg 2013 [** 30 **]**	**Ben-Elia 2011 [** 31 **]**	**Bamberg 2013 [** 30 **]**	**Bamberg 2013 [** 30 **]**	**Bamberg 2013 [** 30 **]**
Intervention arm name	Intervention	Dialog	STEP	Dialog	Dialog	Yeti	Standardised	Standardised	Standardised
Outcome	Public Transport Use (Proportion)	Public Transport Use (summary score)	Walking for transportation (duration)	Walking (summary score)	Cycling (summary score)	Non-driving in rush hour	Walking (summary score)	Public Transport Use (summary score)	Cycling (summary score)
Effect size^1^	0.47	0.41	0.18	0.05	0.03	0.00	−0.05	−0.06	−0.08
**CALO-RE BCTs** ^**2**^									
1		X	X	X	X				
2			X						
5		X	X	X	X				
7		X	X	X	X				
8		X	X	X	X				
10			X						
12		X		X	X				
13						X			
15		X		X	X				
16			X			X			
19			X			X			
20	X	X	X	X	X	X	X	X	X
21	X	X	X	X	X	X	X	X	X
28		X		X	X				
35			X						
40		X		X	X				
**V1 BCTs**									
Persuasive argument		X		X	X				
Pros and Cons		X		X	X				
Boost self-efficacy			X						
**Additional BCTs**									
Contextual constraints		X		X	X				
**Structural interventions**									
Relocation	X								

Presence of a Behaviour Change Technique in an intervention arm is indicated by an X.

^1^Effect size from most effective (greatest increase in alternative, more active travel modes) through to least effective (decreases in active travel modes), where the effect size could be calculated.

^2^1: Provide information on consequences of behaviour in general; 2: Provide information on consequences of behaviour to the individual; 5: Goal setting (behaviour); 7: Action planning; 8: Barrier identification/problem solving, 12: Prompt rewards contingent on effort or progress towards behaviour; 13: Provide rewards contingent on successful behaviour; 15: Prompting generalisation of a target behaviour; 16: Prompt self-monitoring of behaviour, 20: Provide information on where and when to perform the behaviour; 21: Provide instruction on how to perform the behaviour; 28: Facilitate social comparison; 40: Stimulate anticipation of future rewards.

Table 7 suggests that interventions which show larger effect sizes in relation to car reduction are those which include more behaviour change techniques. There is also some evidence that including ‘information on when and where to perform the behaviour’ and ‘information on how to perform the behaviour’ alongside other techniques may be more effective than the other techniques alone. However, it is not possible to conclude whether this is merely related to the association between the increased number of techniques and larger effect sizes. One promising technique may be to ‘prompt generalisation of the target behaviour’; this encourages individuals to generalise behaviour to new settings, so for example if they do not use the car for journeys to work they may be encouraged to no longer use the car for going shopping or to visit relatives. The evidence for other techniques is too limited or is inconclusive.

From Table 8 it is possible to see that more techniques are associated with greater effect sizes, at least for interventions with only behavioural components. Interventions which have both structural and behavioural components may not show the same association, but it is not possible to conclude firmly as only one study with both behavioural and structural components was eligible for inclusion in the review. The results also suggest that providing individuals with ‘information on when and where to perform the behaviour’ and ‘information on how to perform the behaviour’ is not sufficient and would be better combined with other techniques, at least for studies with only behavioural components. Conclusions cannot be drawn firmly for studies with both structural and behavioural components, as only one such study met the inclusion criteria for inclusion the review. There are some promising individual techniques: ‘providing information on consequences of the behaviour in general’, ‘goal setting’, ‘planning’, ‘barrier identification’/’problem solving’, and ‘information and when and where’ and ‘how’ to perform behaviour. However, it is not possible to draw firm conclusions, due to the small number of studies and also because of the association between more techniques and efficacy.

Overall these analyses indicate some useful techniques for future interventions. However, the results must be interpreted with caution due to the exploratory nature of this classification technique and because the evidence relating to intervention efficacy is weak and inconclusive.

## Discussion

### Summary of evidence

This is the first systematic review of the efficacy of behavioural interventions to reduce car use using the most robust available evidence and to apply coding of approach, theory and behaviour change technique of behavioural intervention content. The review includes independent meta-analyses, narrative synthesis, and exploratory analyses to consider relations between efficacy and intervention content. Finally, the review also appraises the methodological quality of the available evidence using Cochrane Collaboration-informed measures.

### Theoretical basis of car reduction interventions

All of the interventions included in this review were developed using a top-down approach; no studies reported user engagement or public patient involvement in intervention development. There was limited evidence that car reduction interventions were theory-based. The results are similar to those found in a recent review which concluded that theory was uncommon in the development of behavioural interventions [43].

### Intervention frameworks of car reduction interventions

Intervention framework analysis identified that majority of the included studies targeted motivation (both reflective and automatic) and psychological capability through: education and knowledge; incentivisation and costs; or enablement and habit reduction. None of the included studies targeted physical capability through training or modelling. Few interventions included in this review targeted opportunity, either social or physical. Opportunity is defined in the Behaviour Change Wheel as the behavioural context, and the authors argue that behaviour in context should be the starting point of intervention development. A lack of consideration of opportunity may contribute towards an explanation of a lack of efficacy of existing behavioural interventions to reduce car use. Combining behavioural interventions with structural changes could help to address this issue in future studies.

### Behaviour change techniques in car reduction interventions

A range of behaviour change techniques were identified in the included studies, although around half of the techniques present in the CALO-RE taxonomy had not been applied in robust studies to reduce car use. The number of techniques present in each intervention arm was on average quite low, particularly since some techniques were present in the control groups, for example ‘prompt self-monitoring of behaviour). The most commonly included techniques were focussed on providing information (for example ‘provide information on where and when to perform the behaviour’) and self-regulation skills (for example ‘action planning’).

### Risk of bias of car reduction interventions

The most robust evidence on car reduction is at considerable risk of bias due to the unclear methodological quality of the individual included studies. This limits the confidence in the conclusions regarding intervention efficacy.

### Efficacy of behavioural interventions to reduce car use

The review suggests there is no evidence for efficacy of the behavioural interventions included in this review to change transport behaviours in relation to decreasing the frequency of car use and increasing the proportion of journeys by more active travel modes. There is insufficient evidence in relation to outcomes such as journey distance and duration which have important implications for health as they imply time spent in sedentary behaviours or physical activity.

The findings from this review are consistent with previous results in the literature. The previous review by Graham-Rowe and colleagues suggested that the evidence in this domain was limited and inconclusive. However, while the previous review suggested that there was potential for behavioural interventions to reduce car use, the current review concludes that there is no current evidence of efficacy. These discrepant findings can be reconciled by acknowledging that the current review only considers interventions which have been assessed using controlled studies. Further, the current review employs meta-analytic techniques, which may be considered more robust in comparison to the narrative synthesis of the previous review. A different review by Ogilvie and colleagues [11] suggested that a 5% shift from car journeys to walking or cycling could be achieved in motivated population subgroups. However, other evidence has been more ambiguous, with the Bird review of walking and cycling interventions [13] concluding that the evidence base was heterogeneous. Further a review of controlled studies of organisational travel plans concluded that there was insufficient evidence for improving health or changing travel mode [44]. The different focus of the reviews and the inclusion criteria can explain some of the variations in the findings.

### Exploratory analyses of relations between efficacy and behaviour change techniques

An exploratory technique, used in previous studies [45,46], revealed that there is some evidence of a relation between study efficacy and the overall quantity of behaviour change techniques identified in an intervention. This result, which was shown in relation to both car reduction and active travel promotion, has been found before in some other domains [46]. There is a suggestion that a promising combination may include the provision of information (‘provide information on where and when to perform the behaviour’, ‘provide instruction on how to perform the behaviour’ and ‘provide information on consequences of behaviour in general’) and behavioural regulation techniques (‘goal setting [behaviour]’, ‘action planning’, and ‘barrier identification/problem solving’). However, these analyses must be interpreted with caution since they are exploratory and based on a very limited number of studies. It is also necessary to acknowledge that there may have been active ingredients, such as self-monitoring, in the control arms in some studies, particularly those utilising alternative intervention arms. This may result in underestimated effect sizes. Effects sizes may also be underestimated in some studies where there are pre-existing baseline differences between the intervention and control participants.

The previous review by Bird and colleagues [13] concluded that the techniques ‘prompt self-monitoring of behaviour’ and ‘prompt intention formation were commonly coded in interventions reporting a statistically significant effect on increasing walking and cycling. These techniques support self-regulatory behaviours and support the findings from this review that interventions to promote more active forms of travel which include self-regulation techniques may show greater efficacy. The current review suggests preliminary evidence however that different individual techniques and patterns of techniques may need to be used to disrupt current behaviours to reduce car use compared to those aiming to promote the uptake of new more active travel behaviours.

### Limitations and recommendations for future research

#### Limitations

One limitation of the review is the exclusive focus on published studies in peer-reviewed journals. It is possible that the effect size estimates would shift with the inclusion of unpublished materials. However, this review aimed to focus on the most robust evidence available and therefore excluded those studies which had not been subject to peer review. This focus was informed by a previous comprehensive review including published and unpublished studies concluded that the strongest methodological studies to reduce car use showed evidence of efficacy. Further, only peer-reviewed articles in English were eligible for inclusion, and this may have resulted in some relevant studies being excluded. A balance between precision and sensitivity in the searches is a characteristic of systematic reviews but may result in a failure to identify eligible studies, however this was counteracted by other additional search methods (e.g., reference searching).

The quality of the included studies in this review is also a limiting factor. The risk of bias of individual studies and across the review is considerable, due to a lack of sufficient reporting. However, this is the most robust evidence available. Meta-analysis is a robust technique for exploring efficacy, but the strength does depend on the included studies which have been identified as poor. There was a lack of common outcome measure in the included studies and therefore some studies were excluded from the meta-analyses. However, the results from all studies are narratively synthesised.

A further limitation is that due to the inclusion criteria it is not possible to draw conclusions regarding the efficacy of structural interventions from this review. While the review included both behavioural only and combined behavioural and structural interventions, only one example of the latter met the inclusion criteria and therefore it is difficult to draw firm conclusions from this. The justification for the exclusion of structural interventions was that that a primary aim of the review was to explore the content of behavioural interventions. The comparison of the efficacy of behavioural and structural interventions remains an important research question.

### Recommendations for future research

Future research should address the issue of the comparative efficacy of structural and behavioural interventions to reduce car use and to promote more active forms of travel. Interventions in this review rarely targeted opportunity and there was limited consideration of behaviour in context. Combining behavioural and structural interventions may allow all components of the COM-B system to be targeted and may result in increased efficacy. Future reviews may wish to use a taxonomy of structural interventions to categorise the content of interventions with structural components to supplement the evidence relating to behaviour change techniques in this review.

A common outcome measure would be beneficial in this research domain. Future studies could focus on outcomes assessing the time spent engaging in sedentary behaviours or being physically active since these have important implications for health. The current evidence is inconclusive with regards to efficacy of behavioural interventions for reducing car journey distance and duration and increasing active travel distance and duration. It is important to acknowledge that even if there is no overall reduction in the number of car trips there can still be important implications for health if individuals decrease the length of journey and therefore the time spent being sedentary. Further, increased periods of being physically active by increasing the distance/duration of active travel journeys could bring health benefits, even without an increase in the overall proportion of active travel journeys.

Existing interventions cannot be described as theory-based and current evidence is limited in terms of theory testing and refinement. Interventions should be developed utilising the existing evidence base, limited as it is, and theory as proposed by the MRC guidance on the development and evaluation of complex health interventions [14]. Future intervention development can be informed by the exploratory analyses which suggest some promising techniques for behavioural interventions. Existing interventions have been developed from a top-down perspective but since the evidence for efficacy is limited and there are suggestions of low compliance future interventions should be developed with the input of potential users. Feasibility and/or pilot studies should be utilised to explore acceptability and feasibility of the intervention and the study protocol. Controlled studies should also employ process evaluation methods to aid interpretation of the results, for example investigating issues relating to intervention delivery, receipt and setting.

The review authors recommend that future research increases the evidence base with high quality, controlled studies with a low risk of bias. Future work should address difficulties relating to blinding of participants in behavioural interventions. There are several ways in which this can be achieved. Disguising the true nature of the intervention may help to achieve participant blinding with limited ethical implications. A procedure that could aid with this purpose would be to separate intervention delivery provider from outcome measurement assessor. The use of objective outcome measures should be included in future studies allowing independent data on behaviour change and avoiding self-report bias. These changes could also be employed to reduce assessor bias, which may be a problem in some existing studies. Future studies should also be mindful of the possibility of existing baseline differences between intervention and control arm participants and control for these. Funders of transport behaviour change interventions should insist on high quality evaluations as an integral part of intervention delivery.

## Conclusions

The review concludes that there is no evidence for the behavioural interventions in included studies to reduce car use frequency. The evidence relating to efficacy of behavioural interventions to reduce car use distance and duration is limited and inconclusive. The overall evidence is highly heterogeneous and also at considerable risk of bias. Future research should investigate alternative behavioural interventions in controlled studies using objectively measured outcomes which relate to sedentary behaviours and physical activity levels. Future studies should be informed by existing evidence, theory, and potential views of potential users. Intervention funders should insist of high quality evaluations of behaviour change trials. Future intervention development can be informed by the exploratory analyses which suggest some promising techniques for behavioural interventions.

## Additional files

Additional file 1:
**Example search terms/strategy.**
Additional file 2:
**Meta-analysis of promoting alternative more active travel modes: Proportion of trips.**
Additional file 3:
**Narrative synthesis of promoting more active travel modes.**
