# Supplementary material for: Efficacy of behavioural interventions for transport behaviour change: systematic review, meta-analysis and intervention coding
Source: Int J Behav Nutr Phys Act. 2014 Nov 28;11:133. doi: 10.1186/s12966-014-0133-9 (PMC4267710; doi:10.1186/s12966-014-0133-9)
Supplement: Additional file 1: — Example search terms/strategy. [file 12966_2014_133_MOESM1_ESM.docx]

# Additional files

**Additional file 1 –** **Example search terms/ strategy**

Example search terms/strategy for PsycINFO database.

| 1. behavio$.mp. | 17. "active travel$".mp. |
| --- | --- |
| 2. habits/ | 18. "multi modal$".mp. |
| 3. intention/ | 19. multi-modal$.mp. |
| 4. exp motivation/ | 20. multimodal$.mp. |
| 5. reasoned action/ | 21. car us$.mp. |
| 6. reduc$.mp. | 22. 9 or 10 or 11 or 12 or 13 or 14 or 15 or 16 or 17 or 18 or 19 or 20 or 21 |
| 7. increas$.mp. | 23. randomi*ed controlled trial.mp. |
| 8. 1 or 2 or 3 or 4 or 5 or 6 or 7 | 24. cluster analysis/ |
| 9. exp transportation/ | 25. cluster$.mp. |
| 10. "commuting (travel)"/ | 26. ("control$ before" adj5 after).mp. |
| 11. drivers/ | 27. field experiment$.mp. |
| 12. driving behavior/ | 28. observation methods/ |
| 13. sustainable development/ | 29. 23 or 24 or 25 or 26 or 27 or 28 |
| 14. traveling/ | 30. 8 and 22 and 29 |
| 15. "active transport".mp. |  |
| 16. "active commut$".mp. |  |
