# Supplementary material for: Efficacy of behavioural interventions for transport behaviour change: systematic review, meta-analysis and intervention coding
Source: Int J Behav Nutr Phys Act. 2014 Nov 28;11:133. doi: 10.1186/s12966-014-0133-9 (PMC4267710; doi:10.1186/s12966-014-0133-9)

**Additional File 2 - Meta-analysis of promoting alternative more active travel modes: Proportion of trips**

Two studies Bamberg 2006 and Ben-Elia [29, 31] are eligible for inclusion in the meta-analysis to investigate the effect of interventions to increase the proportion of more active travel modes. The figure below shows the forest plot of the comparison intervention and control arms in promoting alternative, more active forms of transport. Results with a random effects model show that there is no significant effect of interventions with a standardised mean difference (SMD) of 0.22 (95% CI= -0.23, 0.68) with substantial heterogeneity (*I^2^= 82% and* chi^2^=5.62 [df=1, p=0.02]).There is therefore no evidence for efficacy of the behavioural interventions in these studies to promote the proportion of journeys by more active travel modes. However, the evidence is weak as only two studies are included in the meta-analysis and there was substantial heterogeneity.

[Insert Figure Here]

**Behavioural interventions to increase active travel modes**

Forest plot of random effects model for behavioural interventions to increase active travel modes


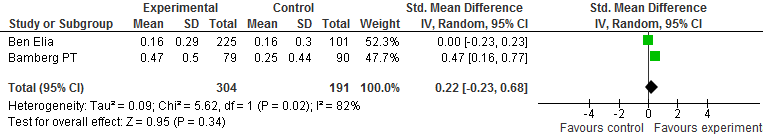

Supplement: Additional file 2: — Meta-analysis of promoting alternative more active travel modes: Proportion of trips. [file 12966_2014_133_MOESM2_ESM.docx]
