# Supplementary material for: Efficacy of behavioural interventions for transport behaviour change: systematic review, meta-analysis and intervention coding
Source: Int J Behav Nutr Phys Act. 2014 Nov 28;11:133. doi: 10.1186/s12966-014-0133-9 (PMC4267710; doi:10.1186/s12966-014-0133-9)
Supplement: Additional file 3: — Narrative synthesis of promoting more active travel modes. [file 12966_2014_133_MOESM3_ESM.docx]

### Additional file 3 – Narrative synthesis of promoting more active travel modes

Narrative synthesis of included studies where the target is the promotion of alternative, more active modes of travel, by outcome.

1. Frequency of trips

Only one included study [39] targeted an increase in alternative, more active travel modes with frequency of trips as the main outcome measure. This study includes 5 different intervention arms: one receiving a free public transport travelcard; one receiving a customised public transport timetable; one receiving a planning intervention; one receiving a free travelcard and customised timetable for public transport; and one receiving a free public transport travelcard in combination with a planning intervention. Effect sizes cannot be calculated for this study due to lack of available data. According to the authors the freetravel card intervention had a significant effect on public transport use in the short term, but this was not maintained in the long term. Further exploration by the authors of the study revealed that the free travelcard intervention increased public transport use significantly, alone and in combination with a planning intervention, but not in combination with a customised timetable. The evidence relating to the efficacy of behavioural interventions to increase the frequency of more active travel trips in limited and inconclusive.

1. Proportion of trips

There are 3 studies relating to increasing the proportion of more active travel modes [29, 30, 31]. The Bamberg 2006 and Ben Elia studies are included in the meta-analysis.

1. Duration of trips

There are 2 studies in the review [27, 37] which include duration of more active travel modes as the main outcome measure. In the Aittasalo study [27] there are 2 arms: a STEP intervention condition and a COMP control condition. The STEP intervention arm receives a 6 month intervention consisting of a group meeting, pedometer provision, logbook, printed materials and monthly emails. The COMP condition acts as a waiting list control and receive the intervention later. In the Aittasalo study there is a small effect (*d*=0.18) increase in the duration of walking for transport following the intervention, comparing the STEP intervention and the COMP control. However, there are pre-existing differences at baseline between the intervention and control in duration of walking for active transport, with STEP participants engaging in fewer minutes per week but increasing throughout the intervention period, compared to the control COMP participants who had a longer duration at baseline but decreased as the study went on. The effects may therefore be underestimated, so some caution should be applied.

In the Mutrie study [37] the intervention group receive a Walk In to Work Out intervention pack comprising of written interactive materials, information, activity diary, and reflective safety accessories. The control condition acts as a waiting list control, receiving the intervention after the 6 month follow up. There is insufficient data available to calculate effect sizes for the Mutrie study.

The evidence relating to duration of non-car journeys is limited and inconclusive

1. Distance of trips

There are no studies relating to distance of non-car trips, therefore the evidence is inconclusive.
